# Betel Nut Chewing Decreased Calcaneus Ultrasound T-Score in a Large Taiwanese Population Follow-Up Study

**DOI:** 10.3390/nu13103655

**Published:** 2021-10-19

**Authors:** Ying-Hsuan Lu, Jiun-Hung Geng, Da-Wei Wu, Szu-Chia Chen, Chih-Hsing Hung, Chao-Hung Kuo

**Affiliations:** 1 Department of Post Baccalaureate Medicine, Kaohsiung Medical University, Kaohsiung 807, Taiwan; u107000007@gap.kmu.edu.tw; 2 Department of Urology, Kaohsiung Municipal Siaogang Hospital, Kaohsiung Medical University, Kaohsiung 812, Taiwan; u9001090@gmail.com; 3 Department of Urology, Kaohsiung Medical University Hospital, Kaohsiung Medical University, Kaohsiung 807, Taiwan; 4 Department of Internal Medicine, Kaohsiung Municipal Siaogang Hospital, Kaohsiung Medical University, Kaohsiung 812, Taiwan; u8900030@yahoo.com.tw; 5 Division of Pulmonary and Critical Care Medicine, Department of Internal Medicine, Kaohsiung Medical University Hospital, Kaohsiung Medical University, Kaohsiung 807, Taiwan; 6 Division of Nephrology, Department of Internal Medicine, Kaohsiung Medical University Hospital, Kaohsiung Medical University, Kaohsiung 807, Taiwan; 7 Faculty of Medicine, College of Medicine, Kaohsiung Medical University, Kaohsiung 807, Taiwan; 8 Research Center for Environmental Medicine, Kaohsiung Medical University, Kaohsiung 807, Taiwan; pedhung@gmail.com; 9 Department of Pediatrics, Kaohsiung Medical University Hospital, Kaohsiung Medical University, Kaohsiung 807, Taiwan; 10 Department of Pediatrics, Kaohsiung Municipal Siaogang Hospital, Kaohsiung Medical University, Kaohsiung 812, Taiwan; 11 Division of Gastroenterology, Department of Internal Medicine, Kaohsiung Medical University Hospital, Kaohsiung Medical University, Kaohsiung 807, Taiwan

**Keywords:** calcaneus ultrasound, betel nut chewing, follow-up, Taiwan Biobank

## Abstract

Chewing betel nut is common in Taiwan. Although previous studies have shown that chewing betel nuts is associated with adverse health effects, findings about the impact on bone density have been inconsistent. Therefore, the aim of this study was to investigate the correlation between betel nut chewing and calcaneus ultrasound T-score in a longitudinal study of 118,856 participants from the Taiwan Biobank. Of these participants, 27,002 were followed up with for a median of 4 years. The T-score of the calcaneus was measured in the non-dominant foot using ultrasound. Multivariable analysis showed that a history of chewing betel nut (coefficient β = −0.232; *p* < 0.001) was significantly associated with low baseline T-score in all participants (n = 118,856). In addition, a long duration of betel nut chewing (per 1 year; coefficient β = −0.003; *p* = 0.022) was significantly associated with a low baseline T-score in the participants with a history of chewing betel nut (n = 7210). Further, a long duration of betel nut chewing (per 1 year; coefficient β = −0.004; *p* = 0.039) was significantly associated with a low ΔT-score in the participants with a history of chewing betel nut (n = 1778) after 4 years of follow-up. In conclusion, our results showed that betel nut chewing was associated with a decrease in calcaneus ultrasound T-score, and thus, it is important to stop chewing betel nut to help prevent an increased risk of osteoporosis in the Taiwanese population.

## 1. Introduction

Osteoporosis is a serious skeletal disease that is associated with clinical consequences including fragility fractures typically involving the wrist, humerus, vertebrae, hip, and pelvis [1]. Fragility fractures can result in persistent pain, disability, economic burden, and increased morbidity [2]. The epidemiological features of osteoporosis are associated with advanced age and female sex [3]. Known risk factors contributing to bone density loss include alcohol abuse, smoking, insufficient physical activity, calcium or vitamin D deficiency, long-term use of glucocorticoids, and low body mass index (BMI) [4,5]. A better understanding of the potential risk factors of osteoporosis would help to prevent the disease. Therefore, it is important to investigate factors associated with an increase in the risk of developing osteoporosis.

According to the World Health Organization, 600 million people chew betel nut worldwide. Betel nut chewing has a long history related to economic and cultural factors in Taiwan [6], where the estimated prevalence is approximately 10%, or about 970,000 adults [7]. However, betel nut chewing is associated with a wide range of diseases including pharyngeal and oral cancer, obesity, metabolic syndrome, neuronal injury, cardiac arrhythmias, myocardial ischemia, sub-mucosal fibrosis, hepatotoxicity, and asthma [8]. These adverse health effects are caused by betel nut-derived alkaloids, specifically arecoline [9]. Arecoline is a bioactive aldehyde that can be detoxified through various metabolic pathways [10]. Nitrosation of arecoline produces nitrosamines such as 3-(methylnitrosamino)propionitrile (MNPN), which has been shown to be a strong carcinogen, inducing tumors in in vivo and in vitro experiments [11,12]. Excess exposure to other betel nut components such as polyphenols and tannins also results in the generation of reactive oxygen species, leading to oxidative stress and inflammatory reactions in cardiovascular and other tissues [13]. Regarding the influence of betel nut chewing on bone, a clinical study of 114 participants revealed that betel quid chewing was associated with a decrease in radiographic alveolar bone density [14]. However, two in vivo studies showed that arecoline and phenolics could act as antioxidants and could ameliorate osteoporosis [15,16]. Therefore, findings on the association between betel nut chewing and osteoporosis are inconsistent, and further studies are needed to elucidate this issue.

Due to the limited data and the lack of a large cohort follow-up study on the association between betel nut chewing and calcaneus ultrasound T-score, the aim of this study was to explore the correlation between betel nut chewing and calcaneus ultrasound T-score in a longitudinal study of around 120,000 participants from the Taiwan Biobank (TWB). Follow-up data were analyzed to elucidate the link between betel nut chewing and changes in calcaneus ultrasound T-score.

## 2. Materials and Methods

### 2.1. Ethics Statement

This study followed the Declaration of Helsinki and was approved by the Institutional Review Board (IRB) of Kaohsiung Medical University Hospital (KMUHIRB-E(I)-20210058). Ethical approval for the TWB was granted by the IRB on Biomedical Science Research, Academia Sinica, Taiwan, and the Ethics and Governance Council of the TWB. All of the participants provided written informed consent.

### 2.2. TWB

The TWB was established to promote health care and chronic disease prevention in response to the aging population and longer average life expectancy in Taiwan. As such, the TWB includes lifestyle, genetic, and medical data on Taiwanese community-based volunteers aged 30 to 70 years of age with no history of cancer [17,18]. After signing informed consent, all of the enrolled volunteers are asked to undergo a face-to-face interview and a physical examination and to provide a blood sample. Body height and weight and BMI (kg/m^2^) are recorded during the physical examination, and personal information, diet, and personal and family medical histories and lifestyle factors are recorded during the in-person interview via a questionnaire.

**Figure 1:**
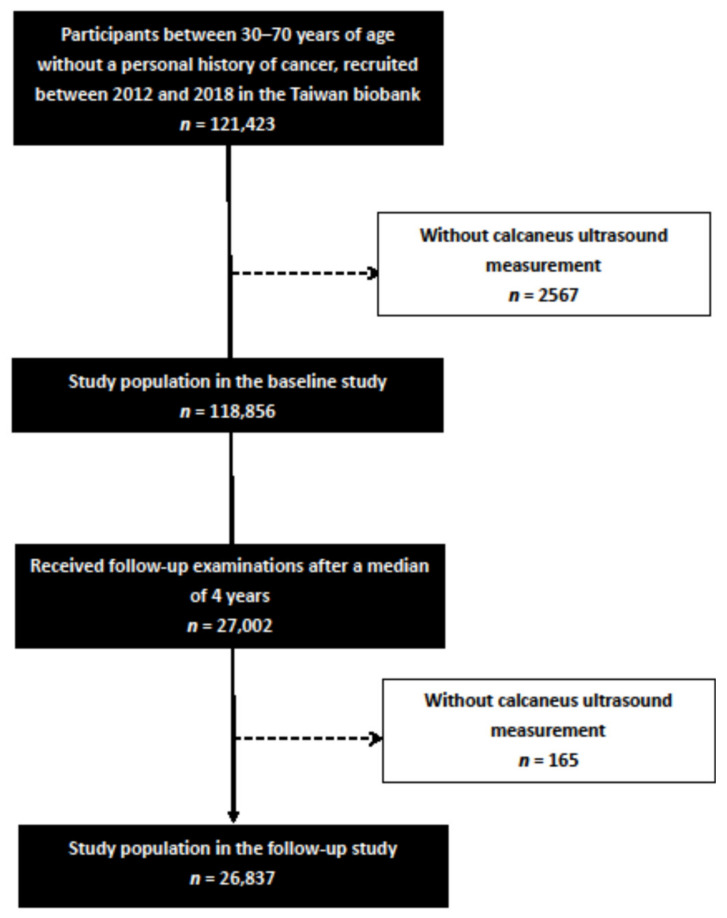
Flowchart of study population.

In this study, 121,423 participants were screened, of whom 2567 were excluded due to incomplete ultrasound data. The remaining 118,856 participants had complete calcaneus ultrasound measurements and were included in the baseline study. Of these participants, 27,002 received follow-up examinations after a median of 4 years. A further 165 participants without complete ultrasound measurements during the follow-up period were excluded, and the remaining 26,837 participants were included in the follow-up study (Figure 1).

### 2.3. Collection of Study Variables

We also recorded baseline variables including age and sex, smoking history, diabetes mellitus (DM) and hypertension. In addition, laboratory data including white blood cell (WBC) count, hemoglobin, fasting glucose, triglycerides, total cholesterol, high-density lipoprotein [HDL]-cholesterol, low-density lipoprotein (LDL)-cholesterol, estimated glomerular filtration rate (eGFR), and uric acid were also recorded. The eGFR was calculated using the 4-variable modification of diet in renal disease study equation: eGFR (mL/min/1.73 m^2^) = 175 × (Scr) − 1.154 × (Age) − 0.203 × (0.742 if female) × (1.212 if African American) [19].

#### 2.3.1. Assessment of Betel Nut Chewing

The participants were asked the following question(s): “Have you ever chewed betel nut?” (“Yes” or “No”) and “How many years have you chewed betel nut?” if they answered “Yes” to the first question.

#### 2.3.2. Assessment of Calcaneus Ultrasound T-Score

An ultrasound device (Achilles InSight, GE, Madison Heights, USA) was used to measure the T-score (g/cm^2^) of the calcaneus in the non-dominant foot. The calcaneus ultrasound T-score was calculated as (T-score of the participant—mean T-score in young adults)/the standard deviation of a normal young-adult population. Change in the calcaneus ultrasound T-score (ΔT-score) were calculated as: follow-up T-score—baseline T-score.

#### 2.3.3. Statistical Analysis

Data are presented as percentages or as means ± standard deviations. The chi-square test was used to analyze between-group differences in categorical variables, and the independent t test was used for continuous variables. One-way analysis of variance was used for multiple comparisons among the study groups. Multivariable linear regression analysis was used to identify associations between chewing betel nut and baseline T-score and ΔT-score. A *p* value of less than 0.05 was considered to indicate a statistically significant difference. All statistical analyses were conducted using SPSS version 26.0 for Windows (SPSS Inc. Chicago, IL, USA).

## 3. Results

The mean age of the 118,856 enrolled participants was 49.9 ± 10.9 years and included 42,658 males and 76,198 females. The participants were stratified into two groups according to the baseline calcaneus ultrasound T score ≥ −2.5 (n = 110,227; 92.7%) or < −2.5 (n = 8629; 7.3%).

### 3.1. Comparison of Clinical Characteristics among the Participants According to BASELINE Calcaneus Ultrasound T Score ≥ −2.5 or <−2.5

A comparison of the clinical characteristics between the participants with a baseline calcaneus ultrasound T score ≥ −2.5 or <−2.5 is shown in Table 1. Compared to the participants with a baseline T-score ≥ −2.5, those with a baseline T-score < −2.5 were older, predominantly male, had longer smoking and betel nut chewing histories, higher prevalence rates of DM and hypertension, higher levels of hemoglobin, fasting glucose, triglycerides, total cholesterol, HDL-cholesterol and LDL-cholesterol, lower BMI, lower baseline T-score, lower WBC count, and higher eGFR.

**Figure 2:**
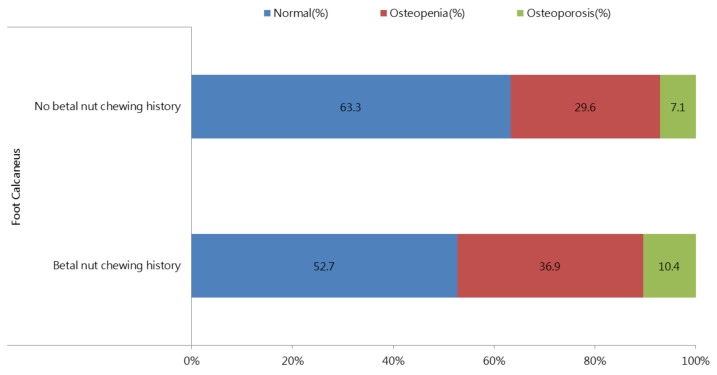
The distribution of calcaneus ultrasound T-score ≥ −1.0, T-score −1.0 to −2.5 and T-score < −2.5, stratified by the betel nut chewing history. There was a significant trend for an increase (*p* < 0.001) in T-score < −2.5 in participants with betel nut chewing.

Figure 2 shows the distributions of those with calcaneus ultrasound T-score ≥ −1.0, T-score −1.0 to −2.5 and T-score < −2.5 stratified by betel nut chewing history. There was a significant increasing trend (*p* < 0.001) in the T-score < −2.5 in the participants with a history of chewing betel nut. The prevalence rates of a T-score ≥ −1.0, T-score −1.0 to −2.5, and T-score < −2.5 in the participants with and without a history of chewing betel nut were 52.7%, 36.9%, and 10.4% vs. 63.3%, 29.6% and 7.1%, respectively (*p* < 0.001).

### 3.2. Correlation between a History of Betel Nut Chewing and Baseline T-Score in All Participants

Table 2 shows the determinants of the baseline calcaneus ultrasound T-score in all of the participants (n = 118,856) using multivariable linear regression analysis. After multivariable analysis (adjusted for betel nut chewing history, age, sex, smoking history, DM, hypertension, BMI, WBC, hemoglobin, fasting glucose, triglycerides, total cholesterol, HDL-cholesterol, LDL-cholesterol, eGFR, and uric acid), betel nut chewing history (unstandardized coefficient β = −0.232; 95% confidence interval (CI) = −0.271 to −0.193; *p* < 0.001), old age (*p* < 0.001), female sex (*p* < 0.001), no DM history (*p* < 0.001), hypertension history (*p* < 0.001), low BMI (*p* < 0.001), high WBC (*p* < 0.001), low hemoglobin (*p* < 0.001), high fasting glucose (*p* = 0.037), high triglycerides (*p* < 0.001), low HDL-cholesterol (*p* < 0.001), high LDL-cholesterol (*p* = 0.021), high eGFR (*p* < 0.001), and high uric acid (*p* < 0.001) were significantly associated with a low baseline T-score. We further performed a false discovery rate (FDR) test, and betel nut chewing history still had significant results (*p* = 0.013).

We further performed subgroup analysis after excluding age older than 50 years in women to survey the association between a history of betel nut chewing and the baseline Z-score (n = 77,676). After multivariable adjustment, betel nut chewing history (unstandardized coefficient β = −0.201; 95% CI = −0.241 to −0.161; *p* < 0.001) was significantly associated with a low baseline Z-score.

### 3.3. Correlation between Duration of Chewing Betel Nut and Baseline T-Score in the Participants with a History of Betel Nut Chewing

Table 3 shows the determinants of the baseline T-score in the participants with a history of chewing betel nut (n = 7210) using multivariable linear regression analysis. After multivariable analysis (adjusted for duration of betel nut chewing, age, sex, smoking history, DM, hypertension, BMI, WBC, hemoglobin, fasting glucose, triglycerides, total cholesterol, HDL-cholesterol, LDL-cholesterol, eGFR, and uric acid), a long duration of chewing betel nut (per 1 year; unstandardized coefficient β = −0.003; 95% CI = −0.006 to −0.001; *p* = 0.022), old age (*p* < 0.001), male sex (*p* = 0.010), smoking history (*p* = 0.010), low BMI (*p* < 0.001), high WBC (*p* = 0.022), low hemoglobin (*p* < 0.001), and high eGFR (*p* < 0.001) were significantly associated with a low baseline T-score in the participants with a history of chewing betel nut. We further performed an FDR test, and the duration of chewing betel nut still had significant results (*p* = 0.044).

We further performed subgroup analysis after excluding age older than 50 years of in women to survey the association between the duration of chewing betel nut and the baseline Z-score (n = 7106). After multivariable adjustment, a long duration of betel nut chewing (per 1 year; unstandardized coefficient β = −0.003; 95% CI = −0.006 to 0; *p* = 0.074) was not but was borderline significantly associated with a low baseline Z-score.

### 3.4. Correlation between Duration of Chewing Betel Nut and ΔT-Score in the Participants with a History of Chewing Betel Nut

Table 4 shows the determinants of the ΔT-score in the participants with a history of chewing betel nut (n = 1778) using multivariable linear regression analysis. After multivariable analysis (adjusted for duration of betel nut chewing, age, sex, smoking history, DM, hypertension, BMI, WBC, hemoglobin, fasting glucose, triglycerides, total cholesterol, HDL-cholesterol, LDL-cholesterol, eGFR, and uric acid), a long duration of betel nut chewing (per 1 year; unstandardized coefficient β = −0.004; 95% CI = −0.008 to 0; *p* = 0.039) was significantly associated with a low ΔT-score. We further performed an FDR test, and duration of chewing betel nut still had significant results (*p* = 0.040).

We further performed subgroup analysis after excluding age older than 50 years in women to survey the association between duration of chewing betel nut and ΔZ-score (n = 1749). After multivariable adjustment, a long duration of betel nut chewing (per 1 year; unstandardized coefficient β = −0.004; 95% CI = −0.008 to 0; *p* = 0.040) was significantly associated with a low ΔZ-score.

## 4. Discussion

In this study, we investigated the associations between chewing betel nut and the calcaneus ultrasound T-score at baseline and follow-up. A total of 118,856 participants were analyzed at baseline, and 27,002 were analyzed after a median follow-up period of 4 years. Overall, we found that a history of chewing betel nut and a long duration of chewing betel nut were associated with a low baseline T-score. Furthermore, after 4 years of follow-up, a long duration of chewing betel nut was associated with a greater decrease in the T-score.

The first important finding of this study is that betel nut chewing was associated with a low baseline T-score, and that a long duration of chewing betel nut was associated with a greater decrease in T-score after follow-up. These results indicate that betel nut chewing plays an important role in increasing the risk of osteoporosis and that the detrimental effect on bone density increases with the duration of use. With regard to the underlying mechanism, preliminary research has reported that betel nut extracts themselves do not alter the expression of osteoprotegerin (OPG), but rather inhibit the expression of alkaline phosphatase (ALP) and stimulate the production of receptor activator of nuclear factor-kB ligand (RANKL) in human osteoblasts [20]. Of these factors, ALP is involved in skeletal mineralization and osteoblastic differentiation, while RANKL binds RANK, which then affects osteoclastic bone resorption [21,22]. In addition, OPG, a soluble decoy receptor, has been shown to stop bone resorption and osteoclast activation by binding with RANKL [23]. However, contrasting results were also reported in an in vivo study, in which betel nut extracts prevented bone density loss by inducing an increase in OPG and inhibiting RANKL expression [15]. The anti-osteoporosis effects have been attributed to arecoline, which has been shown to inhibit the downstream transcription factors nuclear factor kappa B (NF-κB) and mitogen-activated protein kinases activated by RANKL–RANK interactions [16,24]. However, a relatively low concertation of betel nut extract was used in these anti-osteoporosis experiments, whereas a higher baseline concentration of arecoline is found in the saliva of habitual chewers [16,25,26]. Moreover, betel nut is often chewed with a mixture of various ingredients such as tobacco, betel leaf, and calcium hydroxide, traditionally called slaked lime [27]. A previous cross-sectional study reported that Southeast-Asian women with a history of chewing betel nut were associated with an increased bone mineral density (BMD), which may be due to the mixture containing calcium in addition to betel nut [28]. In summary, betel nut has both potential pharmacological applications and toxic effects depending on the exposure dose, duration, and other additives. Therefore, the daily consumption of the hazardous components in betel nut will lead to a cumulative dose that may contribute to osteoporosis by disrupting the balance of molecules such as ALP and RANKL in bone remodeling.

The second important finding of this study is that a high WBC was associated with a low T-score. Increasing evidence has demonstrated that immune responses modify bone remodeling through complex interactions involving neutrophils, lymphocytes, and various cytokines in different diseases and physical conditions such as aging [29]. For example, estrogen deficiency has been shown to induce an expansion of B lymphocytes and T lymphocytes, leading to an increased production of RANKL [30,31,32]. Besides RANKL, an elevation in T lymphocyte-derived tumor necrosis factor-alpha (TNF-α) and the interleukin (IL)-1 family, which act as central mediators in osteoporosis by increasing osteoclast precursors and suppressing osteoblast activity [33,34], has been reported in postmenopausal women and patients with rheumatic diseases [32,35,36]. However, Valderrábano et al. (2017) reported that bone loss was associated with increased neutrophil count, decreased monocyte count, and decreased lymphocyte count in elderly men [37]. In addition, an increased neutrophil lymphocyte ratio has been associated with lower BMD in elderly people with osteoporosis. Aging-related chronic inflammation can also cause an increase in neutrophils and the activation of osteoclasts by RANKL and a wide range of inflammatory cytokines [38]. Taken together, WBCs play a critical role in bone remodeling based on the overlap of pathways between immune activation and bone turnover. Since we only evaluated total WBC count in this study, further studies are needed to investigate the relationship between WBC subtypes and BMD. In addition, our results showed that a high WBC was associated with a low T-score in participants with a history of chewing betel nut. Faouzi et al. (2018) demonstrated that the aqueous fraction of betel nut extracts could stimulate cytokine production, particularly IL-8, in various immune cell lines and human primary immune cells [39]. Since IL-8 induces chemotaxis primarily in neutrophils [40], it is possible that an increased neutrophil lymphocyte ratio may be associated with low T-score in betel nut chewers.

The third important finding of this study is that high levels of fasting glucose, triglycerides, and LDL-cholesterol and a low level of HDL-cholesterol were associated with a low T-score. These findings are consistent with the study by Yamaguchi et al., who reported that both low HDL-cholesterol and high LDL-cholesterol were correlated with low BMD [41]. Bijelic et al. reported that high triglycerides and high LDL-cholesterol but not HDL-cholesterol were associated with bone loss [42]. In addition, Holmberg et al. reported that impaired fasting glucose, an intermediate state between normoglycemia and diabetes, resulted in a small but significant increase in the risk of osteoporotic fractures [43]. Furthermore, hyperglycemia, high triglycerides, low HDL-cholesterol, hypertension, and central obesity are features of metabolic syndrome, which has been associated with osteoporosis via various mechanisms [44]. For example, high glucose levels can lead to the generation of advanced glycation end-products through the nonenzymatic glycation of macromolecules (proteins, lipids, and nucleic acids) and can then induce osteocyte sclerostin expression, which is a negative regulator of osteoblast differentiation [45]. In addition, advanced glycation end-products have been shown to stimulate the expression of peroxisome proliferator-activated receptor gamma (PPARγ) [46], leading to an increase in adipogenesis and a reduction in osteoblastogenesis since adipocytes and osteoblasts share the same progenitors [47]. Increased levels of circulating lipids such as LDL-cholesterol can increase the risk of their oxidation, which then accumulate in the subendothelial matrix of the vasculature and bone. Consequently, oxidized LDL-cholesterol promotes bone resorption by inducing the endothelial expressions of monocyte chemotactic factors and macrophage colony stimulating factor (M-CSF), which then leads to the differentiation of precursor cells into osteoclasts [48]. In contrast, HDL-cholesterol inhibits the oxidation of LDL-cholesterol and facilitates cholesterol transport from vessels back to the liver [49]; therefore, low HDL-cholesterol is associated with low BMD. However, high triglycerides may not be identified as an independent risk factor for osteoporosis since it is often associated with a higher BMI, which is a known protective factor for the skeleton [41]. In summary, alterations in lipid and glucose metabolism are strongly associated with bone remodeling.

Another interesting finding of this study is that a high level of uric acid was associated with a low T-score. Preliminary studies have demonstrated that elevated levels of serum uric acid and gout, a disease characterized by hyperuricemia and inflammation, are associated with an increased risk of hip fractures [50,51]. However, several studies have reported an association between a higher uric acid level and lower bone loss rate [52]. The effect of uric acid on bone is unclear since it has the ability to scavenge reactive oxygen species while also generating free radicals during the process of purine metabolism and uric acid degradation inside the cell [53]. The prooxidant effect of uric acid can promote the oxidation of LDL-cholesterol at a physiological concentration of around 0.5 nM in human plasma [54]. Furthermore, excess uric acid can lead to the generation of proinflammatory cytokines such as IL-1 [55] and TNF-α [56] leading to bone resorption. In conclusion, the role of uric acid in osteoporosis depends on the balance between antioxidant activity and oxidative stress.

To the best of our knowledge, this is the first longitudinal study to examine the association between betel nut chewing and the duration of chewing with the calcaneus ultrasound T-score. The main novelty and strengths of this study are the large-scale investigation and follow-up data. However, there are also several limitations. First, data on the use of medications were not available in the TWB, and certain medications may affect the development or prevention of increased risk of osteoporosis. Second, some information about inflammation, oxidative stress, bone resorption, and formation markers were lacking. Further studies are needed to assess the association between chewing betel nut with inflammation, oxidative stress, bone resorption, and formation markers. Third, the presence of T-score was identified using an ultrasound device rather than dual energy X-ray absorptiometry (DXA). Although DXA is the most commonly used method to measure BMD, quantitative ultrasound does not require the use of radiation, and it is relatively low cost and portable. In addition, a previous study in Chinese women showed that the Achilles InSight system could identify an increased risk of osteoporosis, which had been confirmed by axial BMD using DXA [57]. Finally, all of the participants in the TWB are of Chinese ethnicity, and as such, our findings may not be generalizable to other populations.

In conclusion, in this large follow-up study, we found that betel nut chewing was associated with an increased risk of osteoporosis, and that a long duration of betel nut chewing accelerated the decline in T-score. Our findings suggest that encouraging people to stop chewing betel nut should be taken into consideration for the prevention of an increased risk of osteoporosis in the Taiwanese population.

## Figures and Tables

**Table 1:** Comparison of clinical characteristics among participants according to baseline calcaneus ultrasound T score ≥ −2.5 or <−2.5.

Characteristics	T-Score ≥ −2.5 (n = 110,227)	T-Score < −2.5 (n = 8629)	*p*
Age (year)	49.3 ± 10.9	57.2 ± 9.1	<0.001
Male gender (%)	35.5	41.2	<0.001
Smoking history (%)	27.0	30.1	<0.001
DM (%)	5.0	6.9	<0.001
Hypertension (%)	11.9	17.0	<0.001
Betel nut chewing history (%)	5.9	8.7	<0.001
BMI (kg/m^2^)	24.3 ± 3.8	23.6 ± 3.7	<0.001
Baseline T-score	−0.18 ± 1.49	−3.1 ± 0.49	<0.001
Laboratory parameters			
White blood cell (×10^3^/uL)	5.8 ± 1.6	5.7 ± 1.6	<0.001
Hemoglobin (g/dL)	13.7 ± 1.6	13.8 ± 1.5	0.005
Fasting glucose (mg/dL)	95.7 ± 20.4	98.3 ± 23.4	<0.001
Triglyceride (mg/dL)	115.2 ± 94.6	119.6 ± 86.7	<0.001
Total cholesterol (mg/dL)	195.4 ± 35.7	199.0 ± 37.0	<0.001
HDL-cholesterol (mg/dL)	54.5 ± 13.4	55.1 ± 14.2	0.001
LDL-cholesterol (mg/dL)	120.9 ± 31.7	122.3 ± 32.5	<0.001
eGFR (mL/min/1.73 m^2^)	33.3 ± 46.3	38.8 ± 48.7	<0.001
Uric acid (mg/dL)	5.4 ± 1.4	5.4 ± 1.4	0.114

Abbreviations. DM, diabetes mellitus; BMI, body mass index; HDL, high-density lipoprotein; LDL, low-density lipoprotein; eGFR, estimated glomerular filtration rate.

**Table 2:** Association of betel nut chewing history with baseline calcaneus ultrasound T-score in all study participants (*n* = 118,856) using multivariable linear regression analysis.

Variables	Multivariable (Baseline T-Score)	
	Unstandardized Coefficient β (95% CI)	*p*
Betel nut chewing history	−0.232 (−0.271, −0.193)	<0.001
Age (per 1 year)	−0.056 (−0.057, −0.055)	<0.001
Male (vs. female)	0.473 (0.397, 0.550)	<0.001
Smoking history	−0.001 (−0.024, 0.022)	0.955
DM	0.096 (0.052, 0.140)	<0.001
Hypertension	−0.070 (−0.098, −0.042)	<0.001
BMI (per 1 kg/m^2^)	0.055 (0.052, 0.057)	<0.001
Laboratory parameters		
White blood cell (per 1 × 10^3^/uL)	−0.093 (−0.018, −0.007)	<0.001
Hemoglobin (per 1 g/dL)	0.018 (0.011, 0.025)	<0.001
Fasting glucose (per 1 mg/dL)	−0.001 (−0.001, 0)	0.037
Triglyceride (per 1 mg/dL)	−0.001 (−0.001, 0)	<0.001
Total cholesterol (per 1 mg/dL)	1.022×10^−5^ (−0.001, 0.001)	0.984
HDL-cholesterol (per 1 mg/dL)	0.002 (0.001, 0.004)	<0.001
LDL-cholesterol (per 1 mg/dL)	−0.001 (−0.002, 0)	0.021
eGFR (per 1 mL/min/1.73 m^2^)	−0.010 (−0.011, −0.009)	<0.001
Uric acid (per 1 mg/dL)	−0.014 (−0.022, −0.006)	<0.001

Values expressed as unstandardized coefficient β and 95% confidence interval (CI). Abbreviations are the same as in Table 1.

**Table 3:** Association of betel nut chewing years with baseline calcaneus ultrasound T-score in study participants with betel nut chewing history (n = 7210) using multivariable linear regression analysis.

Variables	Multivariable (T-Score)	
	Unstandardized Coefficient β (95% CI)	*p*
Betel nut chewing years (per 1 year)	−0.003 (−0.006, −0.001)	0.022
Age (per 1 year)	−0.026 (−0.030, −0.023)	<0.001
Male (vs. female)	−0.335 (−0.589, −0.081)	0.010
Smoking history	−0.179 (−0.315, −0.042)	0.010
DM	0.080 (−0.048, 0.208)	0.219
Hypertension	−0.061 (−0.144, 0.023)	0.157
BMI (per 1 kg/m^2^)	0.036 (0.027, 0.045)	<0.001
Laboratory parameters		
White blood cell (per 1 × 10^3^/uL)	−0.036 (−0.054, −0.019)	0.022
Hemoglobin (per 1 g/dL)	0.061 (0.035, 0.087)	<0.001
Fasting glucose (per 1 mg/dL)	0 (−0.001, 0.002)	0.622
Triglyceride (per 1 mg/dL)	0 (−0.001, 0)	0.125
Total cholesterol (per 1 mg/dL)	0.001 (−0.002, 0.004)	0.567
HDL-cholesterol (per 1 mg/dL)	0.001 (−0.004, 0.005)	0.795
LDL-cholesterol (per 1 mg/dL)	−0.001 (−0.004, 0.002)	0.403
eGFR (per 1 mL/min/1.73 m^2^)	−0.004 (−0.005, −0.002)	<0.001
Uric acid (per 1 mg/dL)	−0.015 (−0.038, 0.009)	0.224

Values expressed as unstandardized coefficient β and 95% confidence interval (CI). Abbreviations are the same as in Table 1.

**Table 4:** Association of betel nut chewing years with calcaneus ultrasound ΔT-score in study participants with betel nut chewing history (*n* = 1778) using multivariable linear regression analysis.

Variables	Multivariable (ΔT-Score)	
	Unstandardized Coefficient β (95% CI)	*p*
Betel nut chewing years (per 1 year)	−0.004 (−0.008, 0)	0.039
Age (per 1 year)	0.001 (−0.004, 0.006)	0.754
Male (vs. female)	0.051 (−0.254, 0.355)	0.745
Smoking history	0.032 (−0.169, 0.233)	0.757
DM	−0.092 (−0.278, 0.095)	0.334
Hypertension	0.075 (−0.044, 0.194)	0.218
BMI (per 1 kg/m^2^)	−0.006 (−0.020, 0.008)	0.428
Laboratory parameters		
White blood cell (per 1 × 10^3^/uL)	−0.005 (−0.032, 0.021)	0.688
Hemoglobin (per 1 g/dL)	−0.009 (−0.047, 0.029)	0.638
Fasting glucose (per 1 mg/dL)	0 (−0.002, 0.002)	0.832
Triglyceride (per 1 mg/dL)	−5.358×10^−5^ (−0.001, 0.001)	0.890
Total cholesterol (per 1 mg/dL)	−0.001 (−0.005, 0.004)	0.829
HDL-cholesterol (per 1 mg/dL)	0.003 (−0.004, 0.009)	0.430
LDL-cholesterol (per 1 mg/dL)	6.104×10^−5^ (−0.005, 0.005)	0.980
eGFR (per 1 mL/min/1.73 m^2^)	0 (−0.003, 0.002)	0.715
Uric acid (per 1 mg/dL)	0.029 (−0.006, 0.063)	0.105

Values expressed as unstandardized coefficient β and 95% confidence interval (CI). Abbreviations are the same as in Table 1.

## Data Availability

The data underlying this study is from the Taiwan Biobank. Due to restrictions placed on the data by the Personal Information Protection Act of Taiwan, the minimal data set cannot be made publicly available. Data may be available upon request to interested researchers. Please send data requests to: Szu-Chia Chen, Division of Nephrology, Department of Internal Medicine, Kaohsiung Medical University Hospital, Kaohsiung Medical University.
